# Aspartate transaminase to platelet ratio index (APRI) to assess liver fibrosis in patients with chronic liver disease

**Published:** 2011-06-01

**Authors:** Caterina Anania, Lucia Pacifico, Flavia Ferraro, Eugenia Olivero, Claudio Chiesa

**Affiliations:** 1 Department of Paediatrics and Child Neuropsychiatry, Sapienza University of Rome, Rome, Italy; 2 Institute of Translational Pharmacology, National Council Research, Rome, Italy

**Keywords:** Liver fibrosis, Liver disease, Aspartate transaminase

Dear Editor,

In patients with chronic liver disease (CLD), liver biopsy is currently an indispensable reference method to assess inflammatory activity and fibrosis stage and thus to estimate prognosis and guide management decisions in such patients. Given that the presence of advanced fibrosis is a predictor of nonresponse, the decision [1] to begin antiviral therapy in cases of chronic viral hepatitis is highly influenced by the stage of liver fibrosis. For example, in chronic hepatitis C (CHC) patients, the stage of liver fibrosis is a predictor of response to interferon-based treatment. Despite these premises, liver biopsy has several disadvantages, including poor patient compliance, sampling error, limited usefulness for follow up, and poor intra- and interobservation agreements [2]. Considering these limitations, in the last decade clinical investigators have been searching for noninvasive methods to obtain accurate information about the fibrosis stage in patients with CLD. Accordingly, a noninvasive diagnostic test for liver fibrosis should be simple, available to wide audiences at limited expense, able to accurately identify disease stage, and sensitive enough to track changes in fibrosis induced by the natural course of disease progression or by therapy [3]. Serum biochemical tests, including direct and indirect markers of liver fibrosis, have been the subject of an intensive investigation, but their diagnostic accuracy has been reported to vary widely [3][4]. In this area, of great contemporary interest is the article by Yilmaz et al. on the usefulness of the aspartate transaminase to platelet ratio index (APRI) to assess liver fibrosis in patients with CLD [5]. APRI is a simple and cheap method to ascertain the ratio between AST and platelets and is easy to use in clinical practice. In patients with CHC, APRI performance has been reported to vary widely in diagnostic accuracy (sensitivities between 41% and 91% and specificities between 47% and 95%) for identifying significant fibrosis. A recent systematic review [6] showed that for significant fibrosis, an APRI threshold of 0.5 was 81% sensitive and 50% specific. Furthermore, Cheung et al. found that in patients with CHC, APRI was better in predicting advanced versus significant fibrosis, and the authors suggested that APRIs with a cutoff of 0.5 and 1.5 can be used in routine clinical practice to exclude or identify the presence of advanced fibrosis [7].

Consistent with the previous research, the retrospective study by Yilmaz et al.[5] showed that APRI has an acceptable accuracy for the assessment of liver fibrosis in patients with CHC but not in those with chronic hepatitis B (CHB). Indeed, in Yilmaz et al.'s study APRI was significantly associated with fibrosis scores in subjects with CHC (p = 0.0059) but not in those with CHB (p = 0.1495). The major finding of their study was that the APRI also had an acceptable accuracy for the assessment of liver fibrosis in patients with nonalcoholic fatty liver disease (NAFLD), tending to increase with the degree of fibrosis. In previous observational studies that included patients with NAFLD, the APRI score seldom reached the value of 1 but tended to be higher in patients with advanced stages of fibrosis [8]. In the early stages of NAFLD, patients usually present mild to moderate increases in aminotransferase levels, while platelet counts are usually normal. Fibrosis progresses over time, but it may remain stable for some years. Thus, the focus of the study by Yilmaz et al.[5] is that the APRI may be a useful asset in clinical practice to identify the natural course of NAFLD as it approaches the advanced stages. Nonetheless, much more information is needed on the potential uses of this index in the field of hepatology. More advanced stages of NAFLD appear to be associated with older age, higher BMI, diabetes, hypertension, high triglycerides, and insulin resistance [9]. The findings from different studies are not completely consistent as to which factors (including APRI score) are independently associated with fibrosis progression, and this may depend on the population studied.
